# Supplementary material for: Sub-second temporal magnetic field microscopy using quantum defects in diamond
Source: Sci Rep. 2022 May 24;12:8743. doi: 10.1038/s41598-022-12609-3 (PMC9130321; doi:10.1038/s41598-022-12609-3)
Supplement: Supplementary file 1 — Supplementary Information. [file 41598_2022_12609_MOESM1_ESM.pdf]

Supplementary Information for Sub-second Temporal Magnetic Field Microscopy Using Quantum Defects in Diamond

Madhur Parashar[1,2,†], Anuj Bathla[3,4], Dasika Shishir[3], Alok Gokhale[3], Sharba Bandyopadhyay[1], and Kasturi Saha[3,*]

[1]Information Processing Laboratory, Department of Electronics and Electrical Communication Engineering, Indian Institute of Technology Kharagpur, Kharagpur, West Bengal, India -721302
[2]School of Medical Science and Technology, Indian Institute of Technology Kharagpur, Kharagpur, West Bengal, India - 721302
[3]Department of Electrical Engineering, Indian Institute of Technology Bombay, Mumbai, Maharashtra, India-400076
[4]Centre for Research in Nanotechnology and Science, Indian Institute of Technology Bombay, Mumbai, Maharashtra, India-400076
[†]madhurparashar@iitkgp.ac.in
[*]kasturis@ee.iitb.ac.in

S1.1 Supplementary notes

Normalization of amplitude spectral density: Amplitude spectral density (ASD) of magnetic field traces of individual pixels is defined as the square root of one-sided power spectral density (PSD). One-sided PSD $S(f)$ of a pixel time series X(t) is normalized such that area under the PSD curve integrated on one-side from 0 to $\frac{f_s}{2}$ equals the variance σ_X^2 of the mean subtracted pixel time traces. f_s denotes sampling frequency or frames per second.

$$\sigma_X^2 = \int_0^{\frac{f_s}{2}} S(f)\,\mathrm{d}f \tag{1}$$

The above normalization was implemented with MATLAB in-built functions. For a time series data vector V, the ASD vector is given by

```
fourier_vector = fftshift(fft(V))
ASD = sqrt(2) * abs(fourier_vector) / sqrt(length(fourier_vector))
```

The right half of the ASD vector represents amplitude spectral density from frequency 0 to $f_s/2$.

Determination of single pixel spatial resolution and effective magnification: In the experimental setup, the entire assembly of sample, diamond crystal and microwave resonator was mounted on a motorized XYZ stage and the excitation beam was kept fixed. The motorized stage coordinates are accurate to 100 nm positioning. Magnetic field images of the microcoil sample were acquired at slightly shifted ($\sim$ 20 µm) locations in X and Y motor coordinates. Corresponding to the change in motor coordinates, number of pixel shifts were noted for a sharp feature in the magnetic field image of the microcoil or the microwire sample. The measurements were repeated several times and the per-pixel spatial resolution was evaluated to be 1.33 µm per pixel during the microwire measurements and 1.7 µm per pixel during the microcoil measurements. As mentioned in the main text, the per-pixel resolution during the two sets of sample measurements differ due to slight change in positioning of a focusing plano-convex lens in the excitation-fluorescence collection path of the widefield microscope. The lock-in camera real pixel size is 40 µm, which yields effective magnification of 30$\times$ for microwire measurements and 23.5$\times$ for microcoil measurements.

Per-pixel raw data processing: Additional details to measure raw-data, process and analyze time-dependent magnetic field maps.

1. Before dynamic magnetic field tracking, we acquire a widefield lock-in ODMR spectrum of a single NV resonant feature at high microwave frequency step size of 100 kHz. The resonant feature is selected on the basis of high signal response and linearity at the NV zero-crossing point *i.e.*the NV resonant frequency. This selection determines the NV axis along which the magnetic field sensing will be performed.

2. The informative red light emitted from NV centers spans a limited area on the CMOS array (300 $\times$ 300) pixels. Further, the NV ODMR signal of different pixels differ due to Gaussian nature of optical illumination, spatial non-uniformity of the applied microwave field and limited spot size of the excitation beam. Therefore, it is important to select responding pixels or pixels of interest (POI), see Fig. S3. We define responding pixels as pixels satisfying the following criteria - 1. NV-ODMR response above a minimum threshold. 2. Correct non-linear curve fitting. 3. Falls within a manually marked square area, by visually inspecting the continuity and smoothness of resonant frequency 2D maps of example magnetic field samples. First an average response template is created by taking mean of ODMR response of all pixels, responding and non-responding pixels. Since a high number of pixels are responsive in the ODMR data, the template carries an average ODMR feature. The template is normalized to unit norm and the responses of all pixels are projected onto the template via the dot product. Such projections of all pixels over the unit-norm mean template are scaled between 0 to 1 through division by the maximum projection value across all pixels. Therefore a correlation value is obtained, see Fig. S3(a), for each pixel reflecting the SNR of NV-ODMR signal present in the pixel. Pixels with correlation values higher than a set threshold are selected for further processing. This threshold was kept low at 1e-4 to only reject extremely low SNR pixels at this stage. Fig. S3(b) shows a binary image depicting pixels selected at this initial stage. We observe that a certain area on the 300X300 pixel camera array does not receive fluorescence light, as it is blocked by boundaries of a 45° dichroic mount. Such non-responsive camera pixels are mostly rejected in threshold step. Additional selection of high response pixels occurs with subsequent process of non-linear curve fitting.

3. Non-linear curve fitting is performed to fit derivative sum of two Lorentzian profiles separated by 3.05 MHz to each selected pixel response in the previous step. The MATLAB fit func-

tion `lsqcurvefit` is used to perform a Levenburg-Marquadt non-linear fitting for each pixel ODMR response.

4. Histogram of distribution of resonant frequencies of individual pixels is analyzed. Pixels with artefacts or low ODMR response result in incorrect ODMR curve fits and have widely different resonant frequencies, as much as in gigahertz, from the median resonant frequency. On the contrary, all pixels with sufficient SNR levels have resonant frequencies clustered in a small 'continuous' band near the median resonant frequency (see Fig. S3(c)). This resonant frequency range is visually inspected and provides bounds to the color axis of the resonant frequency maps. This simple bound saturates pixels with wrong curve fits of pixels with low ODMR response and adjusts the dynamic range of the color axis to view sample magnetic field features. After saturating the color axis correctly, sample magnetic field profiles are visible in the resonant frequency 2D maps. Here, we additionally select range of camera pixels (see Square box in Fig. S3(c)) with smooth and continuous resonant frequency variation, high image SNR and presence of sample magnetic field features. Further, sensitivity maps and temporal imaging data are analyzed from these set of responding pixels only.

5. The scaling of raw-data PL intensity data of the pixels to magnetic field time traces was done as described in the main text.

6. In temporal imaging datasets, pixels have an offset value ranging from 0 to 1024 (10-bit scale), but mostly centered in the range of 500-600. Also, when a single resonant frequency is applied, heterogeneous pixels might show different baseline PL value. A small baseline time window of about 250 ms to 500 ms, where no voltage was applied to the sample, was acquired and mean pixel value during the baseline window was subtracted from entire time trace of the pixel. Therefore, all pixels were centered to 0 in the beginning of temporal magnetic field tracking. Further, a 'detrend' in-built MATLAB function was applied over time traces of each pixel to remove linear drifts in the magnetic field tracking data.

Sensitivity versus measurement time: In lock-in detection schemes, the measurement time term in the sensitivity (see Equation 4, main text) is a function of modulation frequency, written as $\tau = (1/\omega_{mod}) * n_{cyc}$. The NV zero-crossing slope also strongly depends on the modulation frequency (see Ref 31 in main text), in stark contrast with non-lock-in detection schemes where the NV slope is independent of modulation frequency. We sweep modulation frequency (to vary measurement time) and measure both NV zero-crossing slope and noise for widefield lock-in magnetometry. Sensitivity versus measurement time is presented in Supplementary Fig. S2. In summary, we note the following trends –

1. NV zero-crossing slope decreases as measurement time reduces or modulation frequency increases.

2. Noise in pixels reduces as the modulation frequency increases from 2.2 kHz to $\sim$ 10 kHz.

3. Sensitivity, a function of both NV zero-crossing slope and noise, does not improve much beyond $\sim$ 15 ms measurement time. For measurement times shorter than $\sim$ 4 ms (250 fps), the nanotesla level per-pixel sensitivity degrades rapidly towards $\sim 1\,\mu T/\sqrt{Hz}$.

S1.2 Supplementary Videos

The video files of imaging datasets in the main text have been uploaded to the supplementary videos.

1. Video1: Imaging video file of data shown in the main text Fig.5. Scale bar 27 µm. Microwire imaging, 1.26 Hz, 78 fps NV acquisition.

2. Video2: Imaging video file of data shown in the main text Fig.6. Scale bar 34 µm. Microcoil imaging, 17.9 Hz, 78 fps NV acquisition.

3. Video3: Imaging video file of data shown in the main text Fig.7. Scale bar 34 µm. Arbitrary waveform dynamics. In this video, each time frame was filtered with a MATLAB function 'filloutliers' to primarily reduce noise in the low-SNR pixels at the edges of the FOV. Outliers from image frames were removed on the criteria of 3 scaled median absolute deviation away from the median. Outliers pixels were filled with nearby local median value.

S1.3 Supplementary figures

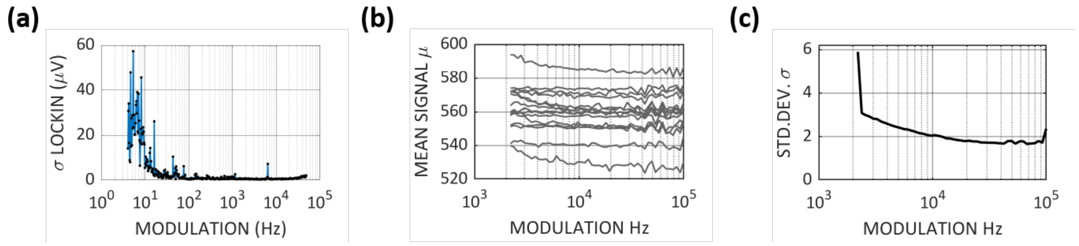

Figure S1: Experimental noise spectrum of widefield imaging setup: (a) Noise spectrum of a reference single photodiode (SP) magnetometry setup across different diamond NV modulation frequency (which is same as lock-in amplifier reference). This spectrum has been measured with all experimental conditions identical to ODMR experiments except applied microwave excitation was off. Low-pass filter time constant set to 100 ms during the measurement. (b) Mean Noise measured for randomly chosen 15 pixels of the lock-in camera measured across different modulation frequencies. Similar to part A, experimental conditions were same as widefield ODMR experiments except microwave excitation was off. Units reflect 1024 (10-bit) points scale of camera output (c) Mean curve of standard deviation of randomly chosen 10000 pixels of the lock-in camera versus diamond NV modulation frequencies. Part (b) and Part (c) data obtained from same set of data of camera lock-in intensity frames ($n = 20$) at different modulation frequencies ($n = 20$ frames collected at each modulation frequency). Units reflected 1024 (10-bit) points scale of camera output. We note that the minimum camera lock-in frequency is 2.2 kHz , and therefore high noise at lower frequencies are not observed, unlike part (a) SP measurements.

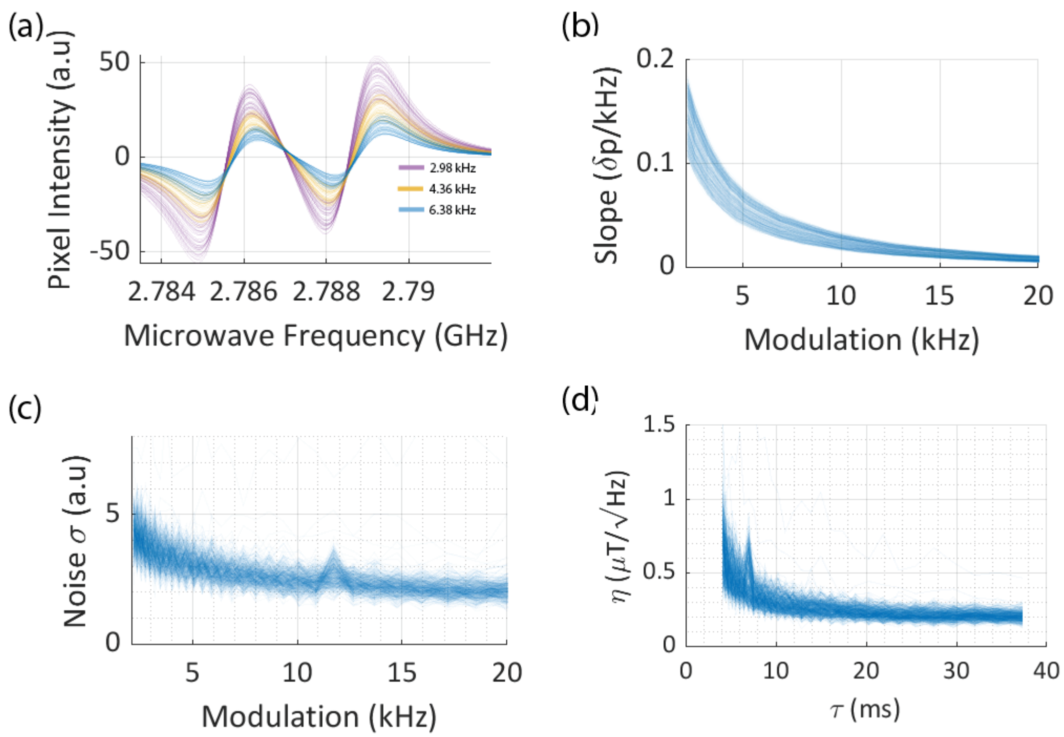

Figure S2: Sensitivity versus measurement time: (a) NV zero-crossing slope decreases with increase in modulation frequency. NV-ODMR response curve, obtained after non-linear curve fits from data, have been shown for few example pixels. Each single trace represents ODMR response of a single pixel. Traces have been color-coded to indicate the modulation (lock-in) frequency. Y axis units a.u represents camera digital output (10-bit) arbitrary units and baseline zeroed. (b) Measured NV zero-crossing slope variation as a function of modulation frequency. Each translucent blue trace represents single pixel, total 400 pixel traces. δp represents change in pixel intensity measured in camera output arbitrary units (c) Measured noise, standard deviation of pixel intensities, as a function of modulation frequency. Each translucent faded trace represents single pixel, total 400 pixel traces. Same example pixels have been used in slope variation and noise variation curve. (d) Measured sensitivity versus measurement time. Each translucent blue trace represents single pixel, total 400 pixel traces. Modulation frequency was varied to get different measurement time intervals, keeping demodulation cycles fixed at 82 cycles.

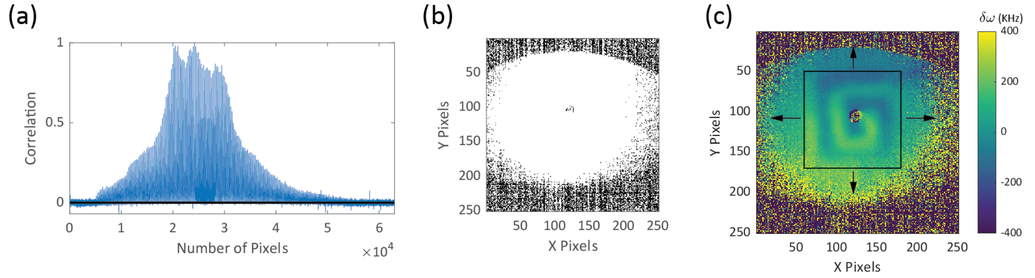

Figure S3: Pixels of interest on camera: (a) Correlation values of NV-ODMR response of each pixel versus unit-norm mean NV-ODMR template. Black horizontal line denotes threshold correlation level 1e-4 used to remove pixels with near-zero ODMR response. During image acquisition of this dataset, response of only 250X250 pixels were stored out of full 300X300 sensor array. (b) Binary image shows selected (white) and rejected (black) pixels based on threshold criteria, as shown in part (a). The curved features in top and bottom part of this image, indicate light blocked by similar curvature boundaries of the 45° dichroic mount in the experimental setup. (C) Resonant frequency map of the microcoil magnetic field showing per-pixel resonant frequency estimates obtained from non-linear curve fits of each pixel's NV-ODMR response data. Pixels, rejected in the part (a), were directly assigned 0 as resonant frequency without performing non-linear curve fits. The black square indicates final selection of responsive pixels, based on visual inspection of resonant frequency map and identification pixels of interest on the full sensor array. Pixels towards the center have high SNR due to Gaussian nature of the 532 nm excitation beam, and therefore, show continuous spatial resonant frequency variations.

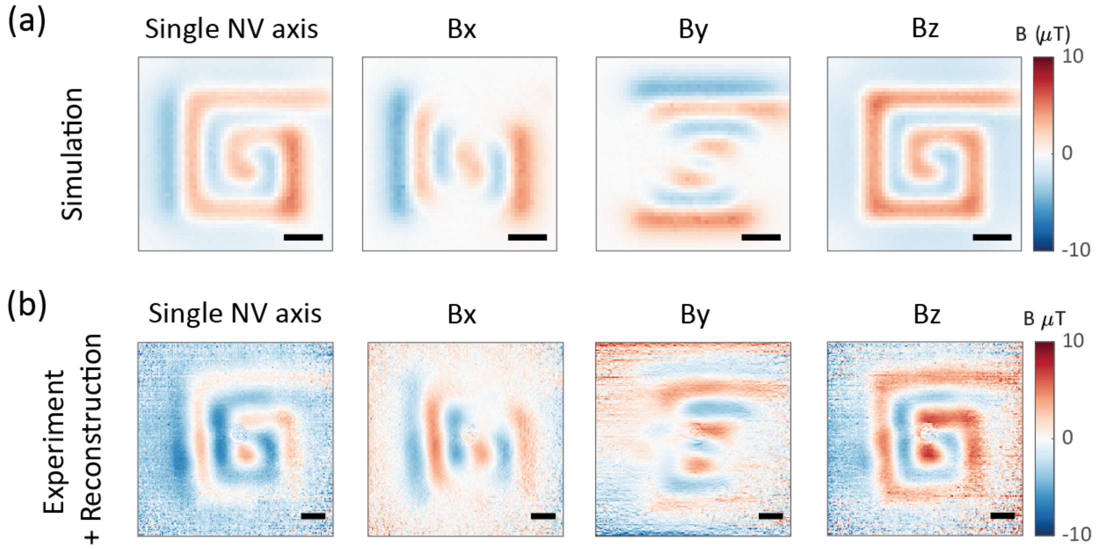

Figure S4: Reconstruction of all three orthogonal axes **B** fields from single NV axis magnetic field image: (a) Simulated magnetic field profiles of the microcoil sample at sample standoff 14 µm, current magnitude 500 µA. The simulated single NV axis projection image has been is shown on the same NV axis about which the widefield ODMR was acquired. Scale bar 40 µm. (b) Experimentally obtained static magnetic field image of the microcoil current flow acquired about a single NV resonance peak with relatively higher magnetic field sensitivity. Orthogonal components of the magnetic field images reconstructed from the single NV axis magnetic image, assuming source free sensor plane and fourier inversion techniques. Scale bar 34 µm

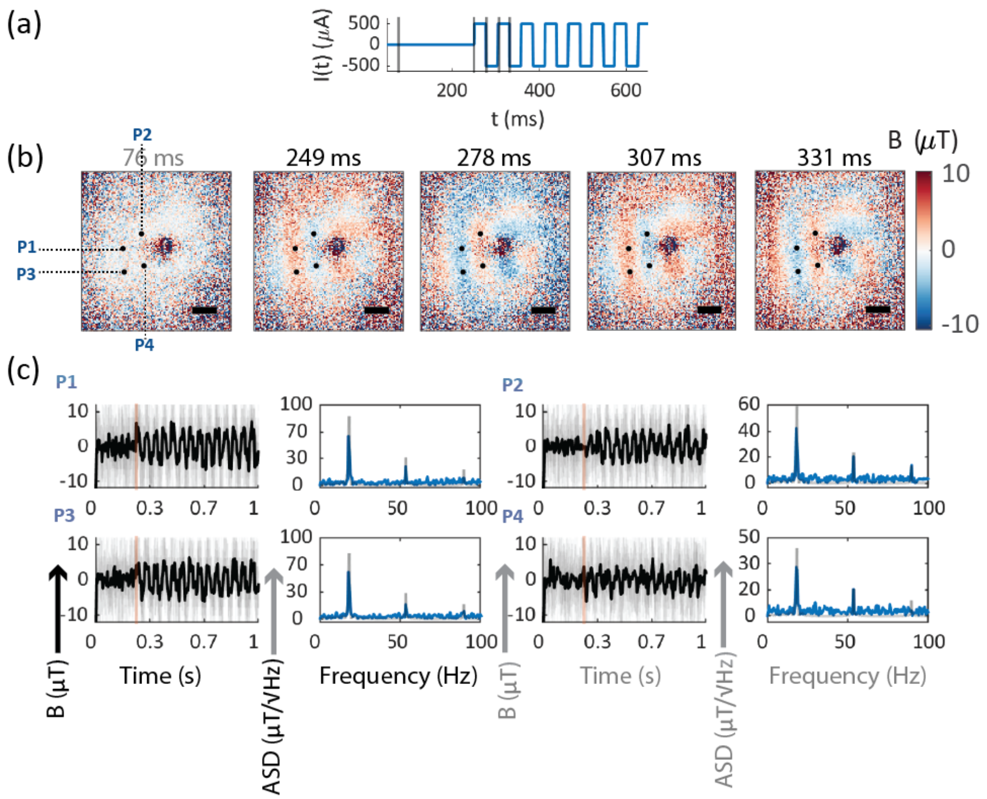

Figure S5: Temporal imaging of 18.9 Hz magnetic field variation at 208 frames per second of the microcoil sample: (a) Applied current waveform (blue line) to the microcoil sample. Black vertical lines mark example timepoints at which single magnetic field frames have been shown. Only few initial example periodic cycles of the applied current waveform have been shown. (b) Magnetic field frames at single time-points (averaged $n = 15$ iterations) showing alternating field image contrast with reversal in current direction. No voltage applied for a baseline time of 0.25 s, first frame selected from baseline window. A periodic square wave voltage waveform of alternating polarity was applied after the baseline time, at 18.9 Hz periodicity and peak current 500 μA. Exact magnetic field frame time-points have been shown on top of each image. Scale bar 34 μm. (c) Full temporal time traces of example pixels showing magnetic field tracking in time. Location of example pixels, labelled P1-P4 in the field of view have been shown in the magnetic field images. For each pixel, magnetic field traces versus time show tracking of applied the magnetic field, with faded gray lines as single iteration traces and solid black lines showing mean ($n = 15$) magnetic field traces for the pixel. Amplitude spectral density of single-pixel field traces are shown on the left, where pixel Fourier spectra are in blue and applied voltage Fourier spectra has been shown in gray. Applied voltage spectral density is scaled to a constant to compare spectral content with pixel Fourier spectra. Since pixels track magnetic field, peaks in the pixel Fourier spectra matches with peaks in the Fourier spectrum of the applied voltage, with peaks occurring at magnetic field variation 18.9 Hz and its odd harmonics.

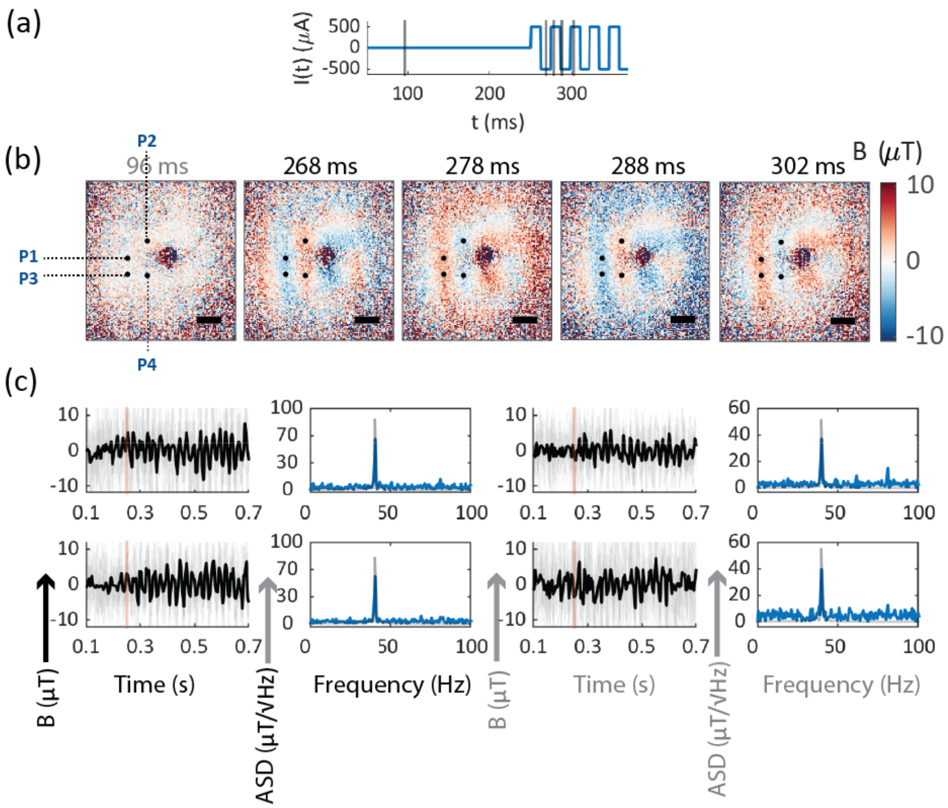

Figure S6: Temporal imaging of 41.52 Hz magnetic field variation at 208 frames per second of the microcoil sample: (a) Applied current waveform (blue line) to the microcoil sample. Black vertical lines mark example timepoints at which single magnetic field frames have been shown. Only few initial example periodic cycles of the applied current waveform have been shown. (b) Magnetic field frames at single time-points (averaged $n = 15$ iterations) showing alternating field image contrast with reversal in current direction. No voltage applied for a baseline time of 0.25 s, first frame selected from baseline window. A periodic square wave voltage waveform of alternating polarity was applied after the baseline time, at 41.52 Hz periodicity and peak current 500 μA. Exact magnetic field frame time-points have been shown on top of each image. Scale bar 34 μm. (c) Full temporal time traces of example pixels showing magnetic field tracking in time. Location of example pixels, labelled P1-P4 in the field of view have been shown in the magnetic field images. For each pixel, magnetic field traces versus time show tracking of applied the magnetic field, with faded gray lines as single iteration traces and solid black lines showing mean ($n = 15$) magnetic field traces for the pixel. Amplitude spectral density of single-pixel field traces are shown on the left, where pixel Fourier spectra are in blue and applied voltage Fourier spectra has been shown in gray. Applied voltage spectral density is scaled to a constant to compare spectral content with pixel Fourier spectra. Since pixels track magnetic field, peaks in the pixel Fourier spectra matches with peaks in the Fourier spectrum of the applied voltage, with the peak occurring at magnetic field variation rate 41.52 Hz.

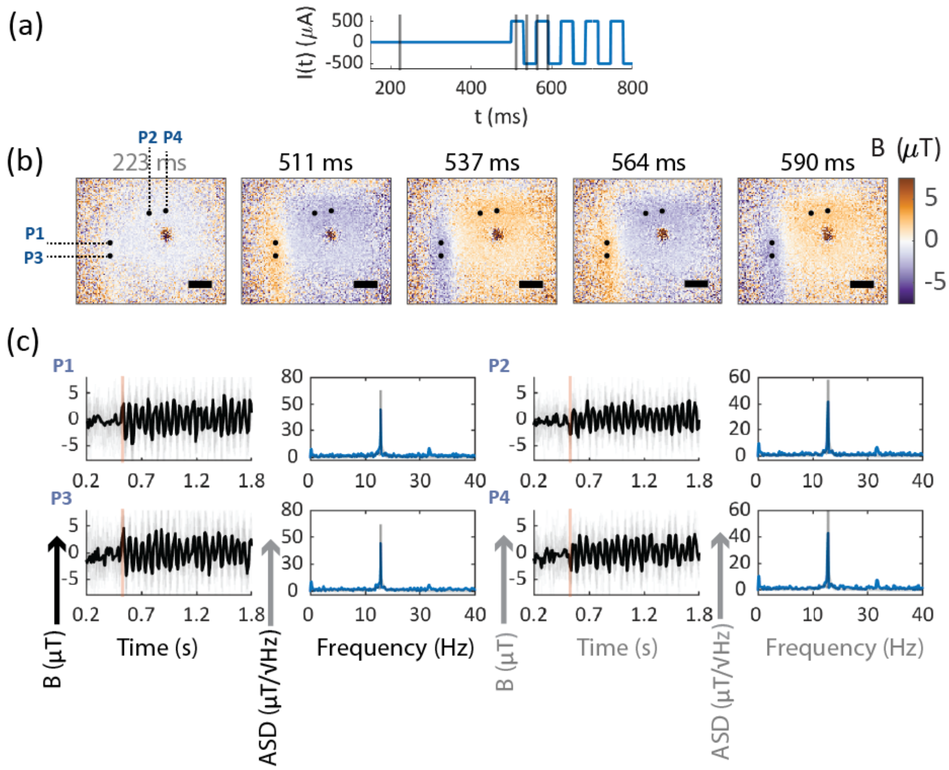

Figure S7: Temporal imaging of 16.3 Hz magnetic field variation at 78 frames per second of the 90° bend microwire sample: (a) Applied current waveform (blue line) to the microwire sample. Black vertical lines mark example timepoints at which single magnetic field frames have been shown. Only few initial example periodic cycles of the applied current waveform have been shown. (b) Magnetic field frames at single time-points (averaged $n = 15$ iterations) showing alternating field image contrast with reversal in current direction. No voltage applied for a baseline time of 0.5 s, first frame selected from baseline window. A periodic square wave voltage waveform of alternating polarity was applied after the baseline time, at 16.3 Hz periodicity and peak current 500 µA. Exact magnetic field frame time-points have been shown on top of each image. Scale bar 27 µm. (c) Full temporal time traces of example pixels showing magnetic field tracking in time. Location of example pixels, labelled P1-P4 in the field of view have been shown in the magnetic field images. For each pixel, magnetic field traces versus time show tracking of applied the magnetic field, with faded gray lines as single iteration traces and solid black lines showing mean ($n = 15$) magnetic field traces for the pixel. Amplitude spectral density of single-pixel field traces are shown on the left, where pixel Fourier spectra are in blue and applied voltage Fourier spectra has been shown in gray. Applied voltage spectral density is scaled to a constant to compare spectral content with pixel Fourier spectra. Since pixels track magnetic field, peaks in the pixel Fourier spectra matches with peaks in the Fourier spectrum of the applied voltage, with each pixel peak occurring at magnetic field variation 16.3 Hz.
